# Improving The well-being of learners with visual impairments in rural Lesotho schools: an asset-based approach

**DOI:** 10.1080/17482631.2021.1890341

**Published:** 2021-03-08

**Authors:** Mamochana A. Ramatea, Fumane P. Khanare

**Affiliations:** School of Education Studies, Faculty of Education, University of the Free State, Bloemfontein, South Africa

**Keywords:** Arts-based methods, asset-based approach, collage, inclusive schools, learners with/out visual impairments, rurality, well-being

## Abstract

**Purpose**: The scarcity of resources, especially in Lesotho rural schools, obstruct the holistic well-being of learners with visual impairments (LVIs). Moreover, the voices of LVIs are often missing about the use of resources to improve their well-being. The study explored an asset-based approach for the improvement of LVIs' well-being in Lesotho.**Method**: Twenty-eight participants, including six teachers, four learners with and four learners without visual impairments, from two rural primary schools in Lesotho, were purposively chosen. Focus group discussions and collages were employed to generate data. The data were then analyzed thematically.**Results**: Involving LVIs in the decision-making, building positive relationships within the school, collaboration with parents and school leaders were provided as 'enabling assets' to improve the well-being of learners. Findings also revealed the constraints on LVIs' holistic well-being, such as, lack of management of the existing resources, shortage of qualified teachers,  and inadequate teaching resources. These were consistent with those identified in the literature, highlighting the asset-based complexity approach in rural schools.**Conclusions**: While the asset-based approach is virtuous, it may not be sufficient if the existing assets are not adequately managed. Therefore, were argue for the critical use of the asset-based approach towards improving the well-being of LVIs.

## Introduction

Globally, inclusive education is considered as a vital tool of addressing and responding to the diversity of needs of all learners through increasing participation in learning, cultures, and communities and reducing exclusion within the education system (Landsberg & Kruger, 2011). There is a growing recognition that learners affected by disabilities should have equal access to primary education that will accord them the opportunity to attain academic goals to their full potential. One such opportunity is that of receiving a free quality education. The high number of learners having disabilities, including LVIs in the world, gives rise to the development, redesigning, and implementation of inclusive policies to assist in providing access to education for all. For instance, although Lesotho is a proponent of inclusive education and primary education is described as a constitutional right in the Children’s Protection and Welfare Act (7 of 2011) and the Education Act (3 of 2010), the holistic well-being of LVIs in rural primary schools and using assets-based approach is still limited. Hlalele (2012) and Martin (2015) note that rural communities, including rural schools, are often isolated and often lack resources, meaning that these communities could struggle. LVIs in these rural communities are made vulnerable due to inadequate support from untrained teachers (Matlosa & Matobo, 2007; Urwick & Elliott, 2010). The lack of resources in rural schools puts children at risk of receiving an inferior education. This is equally true in the case of LVIs. This exacerbates their vulnerabilities (Moloi et al., 2008; Mosia, 2014). The existing perspectives on rural communities and rural schools often do not regard rural communities or rurality as evolving, generative, and sufficient (Balfour et al., 2008). However, in African countries such as Lesotho, rural communities and rural schools are essential resources by having input in the country’s finances and adding support to the holistic development of children, including LVIs in primary schools.

The role of rural communities and rural schools as change agents in pursuit of the holistic well-being of their communities or society as a whole is well documented in (Argall & Allemano, 2009; Moletsane, 2012; United Nations Children’s Fund [UNICEF], 2010). However, when such communities fail to participate fully, this may affect their needs (Mueller, Alie, Jonas, Brown & Sherr, 2011) and are at high risk of been seen as only “receivers” of donations from external suppliers. In this study, the success for the improvement of the well-being of LVIs in rural Lesotho schools is in line with the study of Butler and Mazur (2007) that the community success depends on the role played by local people in rural areas and how they adapt to sustain their lives. Therefore, improving the well-being of LVI in rural primary schools will depend on understanding rural community work (Butler & Mazur, 2007; Mahlomaholo, 2012). This will also depend on how people in rural communities or schools use their assets because they know their own lives and contexts, and drawing from this may contribute to improving the well-being of the LVI, their schools or communities.

Assets or resources seem to vary cross-culturally due to differing expectations. They vary between Western and African countries and between urban communities and rural communities, the reason being that rural communities lack behind (Hlalele, 2012). Living in rural areas appears to be related to fewer resources (Moletsane, 2012). However, Nel (2017) posits that an asset-based approach is a community development strategy that primarily recognizes and strengthens the assets of individuals, associations, and institutions based on perceived local needs. Mansvelt (2018), and Myende (2013) concurs, the findings that rural communities and schools affirm and acknowledge positive actions and practices by building on what is available rather than what is missing.

Since rurality may be linked to a specific culture, it is of interest to understand how an asset-based approach could improve the well-being of LVI in primary schools in Lesotho, since this phenomenon may be pertinent to their lives. Antithetically, rural communities are popular for the strong support emanating from their close-knit family structures. These communities believe and appreciate that individual members have assets, resources, skills, abilities and gifts to share, and solve problems (Kretzmann & McKnight, 1993). There is growing recognition of an asset-based approach as a move from deficit paradigm, refocusing on people’s strengths, abilities and capacities (Kretzmann & McKnight, 1993). Hence, the rural schools, including learners need to be consulted about their needs, as their own interpretations provide important insight into many contemporary issues, including school environments that enable their well-being. An asset-based approach challenges problem-oriented approach that is often applied by research, especially with rural contexts. For instance, an asset-based approach is intended to help individuals and organizations identify, mobilize and harness the existing assets to solve the problems and support participation and collaboration (Ebersöhn & Eloff, 2006) before seeking help from external sources. Therefore, researchers acknowledge that the asset-based theory does not reject the support from other institutions or external communities, especially in rural communities, still faced with challenges supporting vulnerable learners.

Although the existing literature discusses extensively the plight of vulnerable learners in general, there is paucity of literature on improving the well-being of LVIs by using an asset-based approach in rural schools. In particular, the aim of the current study was intended to focus on the use of an asset-based approach that could improve the well-being of LVIs in two primary schools in the Berea district in Lesotho. This study will, therefore, contribute to filling this gap and providing a better understanding of the use of an asset-based approach towards improving the well-being of LVIs in primary schools in the Berea district of Lesotho. Because this was part of a Master of Education dissertation, this article only reports on the data generated during the second and third stages of data generation process, which was to determine factors needed to improve the well-being of LVIs in a primary rural school by answering the research question, *what factors enable and constrain the use of assets to improve the well-being of LVIs in primary schools in the Berea district of Lesotho?*

## Methodology

### Design

This study involves a qualitative approach and a transformative paradigmatic stand (Cohen et al., 2011) as a preferred approach in exploring the use of an asset-based approach to improve the well-being of LVIs. Qualitative research approach involves understanding the participants’ points of view under investigation (McMillan & Schumacher, 2010). This qualitative research is, therefore, conducted using an arts-based research design as it is valued for its ability to reveal rich data by incorporating the voices of multiple participants (Simmons & Daley, 2013). An arts-based research design was employed in this study to allow the teachers, LVIs and LWVIs at selected rural primary schools to express their views within their natural settings with their unique assets or resources since they are experts and assets in their own right.

### Participants

Data was generated from May to October 2019 in two rural primary schools located in the Berea district of Lesotho, where LVIs receive teaching and learning. These schools offer mainstream education to all learners with different disabilities which predominantly consist of learners without any impairment, followed by LVIs, learners with intellectual disabilities and others, however, are on the other hand challenged to respond adequately to the unique needs of such learners (Khanare & De Lange, 2017). Twenty-eight participants were chosen through purposive sampling. The LVIs/LWVIs, aged between 10 and 13 years, starting from grade 4 to grade7 of both sex (both girls and boys) and the teachers both males and females, in particular, those with three and above years’ experience of teaching learners with SEN including LVIs participated in this study as having relevant and required information (Maree, 2017) about the improvement of the LVIs’ well-being. From an asset-based approach, both LVIs and LWVIs can learn to share ideas and resources and be agents of their own well-being. Moreover, because the learners usually challenge the existing systems and policies (Mathikithela & Wood, 2019), it was vital that teachers are involved in the study to hear the learners’ views, and act as agents to ensure that the learners’ voices are heard and inform decision-making in the school context.

### Data generation and procedures

This article reports on the findings of the second and third phase of data generation process that aimed to explore school assets that could improve the well-being of LVIs in the rural primary school. Focus group discussions (Nyumba et al., 2018; Yin, 2017) employed in this study enabled participants to share ideas, discuss together what they perceived to be assets or resources that could be used to improve the well-being of LVI. Within this method, probes and prompts were used to elicit rich information from the participants (Yin, 2017) were engaged in the discussions about how these assets could be used, to what extent were these assets used and how could these assets be enhanced to improve the well-being of LVIs in Lesotho rural primary school setting.

Data relating to identifying available assets for improving the well-being of LVIs were generated over two months in 2019 at two rural schools. In each school, two focus group discussions were conducted as follows: one for learners (LVIs and LWVIs) and one for the teachers. Each focus group discussion lasted for about an hour. The idea of separating learners from the teachers was to ensure that learners do not feel coerced by the teachers’ presence and feel free to interact and share their ideas. Participants were allowed to talk with minimal interruption. Probes and prompts were used to elicit deeper information about the assets, and learners were also encouraged to interact with each other by asking questions, making comments or seeking clarifications (e.g., tell me more about the assets, how the assets mentioned will help the LVIs).

Following the focus group discussions, the next phase of data generation was done through collage method (Mitchell, 2011). Again the collage activity took place over two months in 2019 at the two rural schools. The collage was used to elicit a deeper understanding of the assets that were identified during the focus group discussions and were expanded when appropriate. Some of the key questions were: *What factors could enable the existing assets to improve the well-being of LVIs. Are there certain constraints to using existing assets that feel need to be identified and improved?*

Since none of the participants had used collage or had training in Collage, we engaged the participants in a brief collage training by drawing on the practical examples and suggestions of other arts-based researchers (e.g., Butler-Kisber, 2010; Mitchell, 2011; Simmons & Daley, 2013). In each school, one training was done for the learners and the other one for the teachers. The collage training was guided by questions related to the following topics: (1) definition of a collage, (2) creating a collage, (3) showing examples of previous collages, and (4) sharing ideas about what is and what is not in the collage in relation the broader needs of learners in the schools. Then, participants were allowed to create their own collages in response to the following prompts: *Create a collage showing how school can improve the well-being of LVIs. Your collage can be in a form of words, drawings or pictures taken from the magazines*. To create collages, the researchers adopted the three-session process. First, the participants were given collage materials (e.g., magazines, glue, A4 size and A3 size papers, pens, pencils and scissors). They were allowed time to brainstorm, identify and write all their issues on an A4 size paper. Second, the participants were instructed to look on their list then create their collages using pictures, words or symbols. Third, the participants displayed their collages on the wall and explained what is in their collages to the rest of the other learners, teachers and the researchers. Collages included many words/phrases to be analysed however, this session enables the researchers to elicit a deeper understanding of the assets each group identified and how could they be used to improve the well-being of LVIs. In total, four collages were produced creating space for teachers and learners to share their ideas in both written, spoken and using pictures, therefore leaving no one behind in communicating their ideas. During their presentations, the groups generated a collaborative understanding of what they felt constitutes assets that could improve the well-being of LVIs within the school environment.

**Figure 1.**
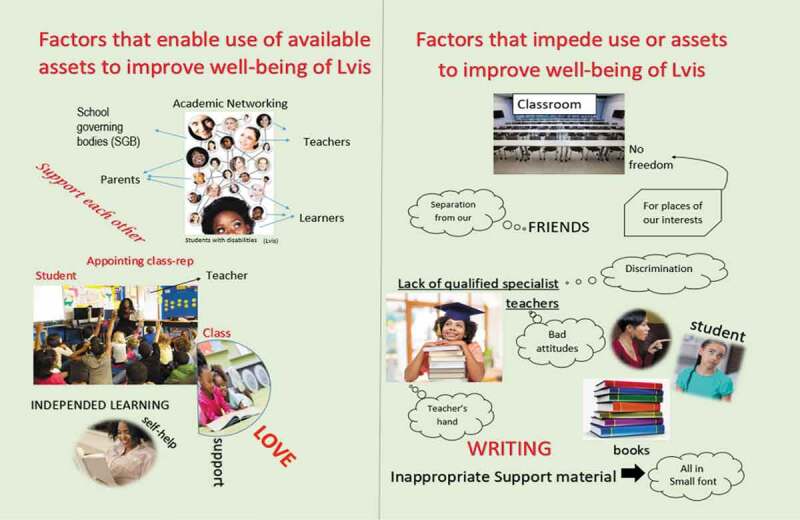
School A, learners’ collage

**Figure 2.**
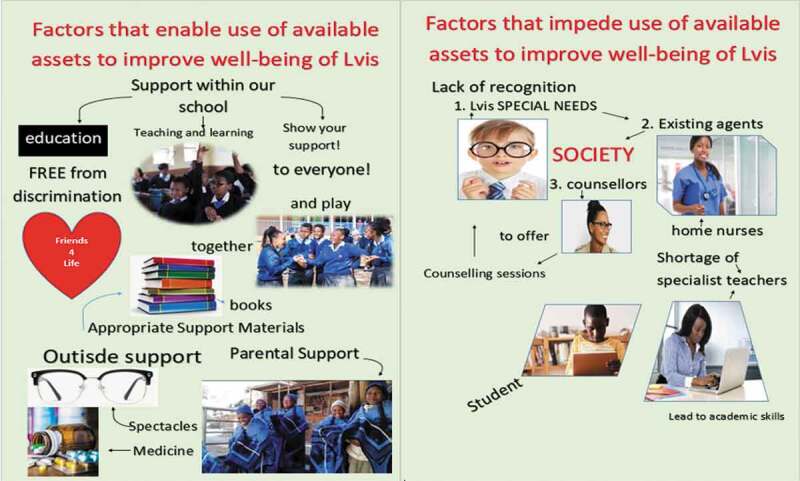
School B, learners’ collage

**Figure 3.**
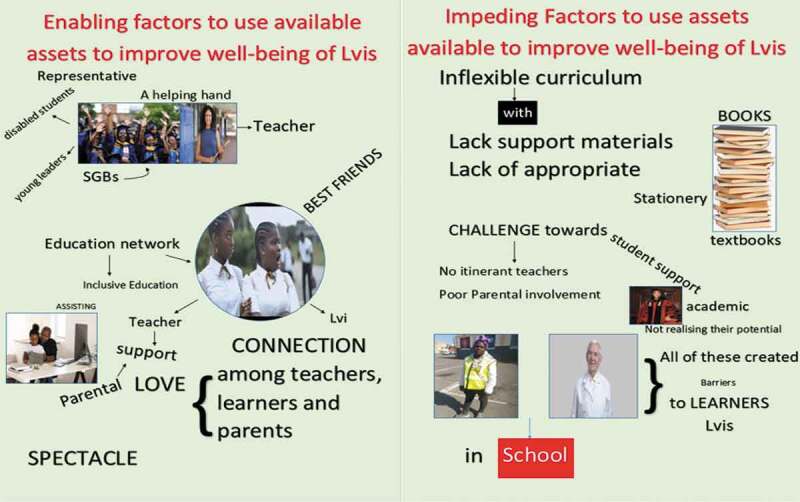
School A, teachers’ collage

**Figure 4.**
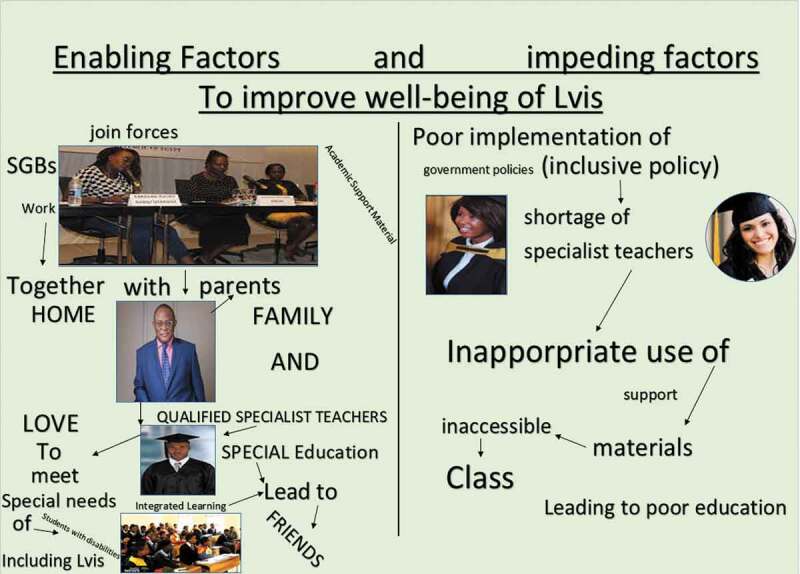
School B, teachers’ collage

Figures 1–4 *present an overview of the four collages that emerged from the study*.

The collage process works very well with the participants; pictures in their collages became a catalyst to trigger more questions, and seek more clarity in order to provide a collective rather than an individual view (Simmons & Daley, 2013). The advantage use of collage in generating data was; it is an age appropriated method for the participating learners involved in this study to enable them fully contributed their ideas (Mayaba & Wood, 2015). Collages created space for teachers and learners to share their collages, which resonates with the theoretical underpinnings of the study, and includes the view that every individual has potential, abilities, or assets (Ebersöhn & Eloff, 2006). Finally, their responses (spoken and discussions) were video-recorded together with written words from the collages were later transcribed verbatim, categorized according to attributes concerning improved well-being of LVIs within the school environment. This study researched the phenomenon (Maree, 2017) on the ways in which resources in rural schools could improve the well-being of LVI through the asset-based approach. The phenomenon was explored through focus group discussions and collages with learner and teachers for crystallization as described by (Ellingson, 2009).

### Data analysis

Textual data (spoken, written and discussion) were generated from the participants’ responses during focus group discussions and collages. The data were analysed through conventional thematic analysis technique. There are several approaches to thematic analysis and we adopted guidelines recommended by Braun and Clarke (2006). According to Braun and Clarke (2006, p. 6), “thematic analysis is defined as a process of identifying patterns or themes within qualitative data.” Braun and Clarke recommends: familiarization with the data, identifying significant codes (e.g., related codes were grouped using colour codes), formulating meanings, clustering themes, developing an exhaustive description, producing a fundamental structure and seeking verification of the fundamental structure as the processes in analysis.

Driving a thematic analysis in this study was that transcripts from the direct quotations of participants’ focus group discussions, words and discussion during collages presentations were subjected to iterative reading (McMillan & Schumacher, 2010). As very little is known about the asset-based approach in relation to improving well-being of LVIs in the literature, we adopted an inductive approach in data interpretation. An inductive approach to analysis suggests that themes identified are generated from the data sets (Braun & Clarke, 2006; Clarke & Braun, 2013; Creswell, 2014). Two researchers (Ramatea and Khanare) met severally to harmonize the findings in order to ensure the participants’ information about using an ABA to improve the well-being of LVIs within their selected schools.

Permission to undertake the study was obtained from Berea Ministry of Education and the university in question. Informed consent was obtained from the school principals, teachers, parents, guardians, and assent from the learners. Participation and pseudonyms were used to ensuring participants’ anonymity and confidentiality. We gained permission from the participants whose collages are reported on here.

## Ethical considerations

Research ethics remain an important consideration in any research, for it ensures confidentiality and safety of the participants, the researchers, and challenge any unacceptable human behaviour (Resnik & Shamoo, 2015). For this reason, the entire process of research mandates the researcher to protect the participants from any harm that may occur as a result of the research process (McMillan & Schumacher, 2010). To adhere to this custom, necessary ethical issues were taken into consideration. University of the Free State under the Faculty of Education Ethics Review Committee (Ethical clearance number UFS-HSD2019/0093/0205) granted the researchers ethical clearance for the study. Permission was also granted by the Ministry of Education and Training in the Berea district and the school principals in Lesotho. Moreover, consent by the teachers as well as assent from the learner themselves were sought and granted. Both the consent and the assent letters were made to ensure that the participants understand the details of the entire research process before the study commences. In this particular instance, the consent and assent forms were written and explained in the participants’ first language (in this case Sesotho). Also, a bigger font was used for LVIs to ensure the fairness of the ethics process (Creswell, 2014). The participants were also made aware of their right to participate or withdraw at any stage of the research (Cohen et al., 2011). During the data generation methods (Focus group and collage discussions), the researchers ensured that the rights of the participants were not violated throughout the research process (Cohen et al., 2011). Pseudonyms were used throughout the study to ensure anonymity and any potential harm (Bertram & Christian, 2014). Therefore, the researchers drew from a number of universal ethical principles, such as, Articles 3 and 9 of the Universal Declaration on Bioethics and Human Rights (UNESCO), the declaration of Helsinki (World Medical Association, 2013), and ethical approaches to gathering information from children and adolescents in international settings (Schenk & Williamson, 2005) to ensure that the principles of human dignity, human rights, privacy, and confidentiality were adhered to.

## Findings

This study values and appreciates the active and collaborative role that young people with or without visual impairments, as well as their teachers play in improving their well-being in a rural school context, by employing the lens of an asset-based approach. The findings are presented in three sections: first, by detailing the participants’ perceived enabling factors with the rural school context, second, by describing the perceived factors constraining in the rural school context, and third, by describing how the participants envision improved well-being of LVIs in a rural school context.

## Enabling factors within the rural school

The participants often described that various factors could enable the usage of assets within their rural schools to improve the well-being of LVIs. The participants referred to LVIs’ involvement in decision-making and strengthened relationships.

### LVIs’ involvement in decision making

The involvement of LVIs in the decision-making was found significant for their holistic well-being. Many participants were mindful of the role of LVIs and created a space to realize their potentials, listen to their problems and challenges, and offer to help to meet their needs appropriately. These showed being part of the decision by LVIs as a factor that enabled them to become assets in their own right. Evidence of the above claims is relevant to what other participants in this study suggested and how the participating LVIs felt; they had a unique capacity, skills, and talents that people from their local community should develop on and capable of making their own decisions. These included the following: having the freedom of movement in the class, having a say in the issues concerning them, becoming class representatives, and independent young leaders. The following quotations indicated and highlighted how the LVIs’ involvements become enabling factors; Learner 6 from school B explained: “. … . *Learners are given a chance every beginning of the year to discuss on sitting plan and appoint class reps*”, Learner 4 from school A: elaborated more on this point in referring to their collage on [Figure 1] by saying: “*In our collage here, learners are appointing class representatives, this enables them including LVIs to take part in the decisions concerning during school meetings”* Teacher L from school A: highlighted the importance of appointing class reps by saying: “ *… Having learner’s representatives in the monitoring of a class is a promising step because the involvement of learners in the issues concerning them enhances the school system and opens doors for the voices of all learners, including LVIs”. The experience of Teacher M*, within the same school, was similar and further explained by pointing to their made collage in Figure 3: “*Engaging learners as class representatives in sharing of ideas and offering helping hands help the school to address and meet the needs of all learners appropriately. Moreover, help such learners to become independent young leaders.”* The participants’ responses seemed to recognize role played by LVIs in decision-making processes. There was no obvious difference in the responses of learners and teachers—both appeared to be equally likely to involve LVIs for support.

### Strengthened relationships

The secure connection among learners, teachers, and parents was seen as a resource on which the participants draw towards the improvement of LVIs’ well-being. The LVIs were encouraged and appreciated the support received within their school towards the improvement of their well-being. A healthy relationship among various stakeholders (learners, including LVIs, teachers, and parents) was seen as the enabling factor. This was able to help them build relationships and enable academic networking within their schools. Learner 2 from school A excitedly said: *“We have decided to put different important people being; (teachers, parents, and learners) as assets in our school,” pointing at their collage* [Figure 1]. *“As learners in my school, we supported each other regardless of the disabilities.”* Teacher S: *“Yes, the connection among parents, learners, and teachers shows that the support is not only needed from teachers but form teamwork” Teacher S also referring to collage* [Figure 3]. An appraisal of both the teachers and the learners’ responses reveal the parents in the rural areas as resourceful through which they could connect with to strengthen the school’s existing relationships. Thus, by identifying assets outside the school environments, both teachers and learners are capable of improving the well-being of LVIs by drawing from resources other than teachers and peers alone. Shared relationship towards children nurturing is a dominant characteristic in the rural areas. So far, the findings indicated how the parents play an essential role in the development of successive inclusion programmes. Hence, parents, teachers and learners support was very vital for the improvement of the well-being of LVIs because this view aligns with the African worldview as expressed in the Sesotho proverb *“lets’oele le beta poho*” (simply translated as a “crows defeat a bull”) (Sefotho, 2018).

## Constraining factors within the rural school

In the process of improving the well-being of LVIs, several issues were identified as constraints on the use of available assets to improve the well-being of LVIs. These included a shortage of qualified specialist teachers, unavailability of appropriate support materials, which resulted in inappropriate usage of classroom teaching and learning resources, and inaccessible school classrooms.

### Shortage of qualified specialist teachers

The perceived shortage of specialist teachers was negatively related to a lack of recognition of LVIs’ holistic well-being, in this case, LVIs/SEN. The factors relating to shortage of specialist teachers are still evident in some of the participants’ current experiences, thus: specialist teachers’ work-loaded and lack of appropriate skills among general teachers, which in turn excludes some of LVIs’ unique needs. Learner 7 from school A, stated: *“Shortage of specialist teacher created challenges to LVIs because, in some classes where there are no specialist teachers, teachers neglected LVIs’ needs and required them to do things like the normally seeing learners”* Similarly, another learner added a comment denoting her experience; Learner 2 from school B: “ … *the other problem of having a shortage of specialist teachers as shown in our collage here, pointing on collage* [Figure 2] *is lack of recognition of our different SEN”*. Teacher S, referring to their college [Figure 4] said: *“The problem of having less specialist teacher ratio in our school created a workload to such teachers as they are be expected to teach in their classrooms, on the other hand, helping other general education teachers with appropriate skills needed to assist learners with SEN including those with eye problems*.”

### Unavailability of appropriate support materials

Lack of appropriate support materials posed as a significant threat that negatively affects LVIs’ learning and consequently affects their well-being. One of the participating teachers spoke with intense feeling stressed that: *“ … .The Lesotho education stakeholders (curriculum designers and Ministry of education and training) are not doing what is expected from them. I do not think they are in line with the intentions of the inclusive education system if textbooks delivered for LVIs are in such small font, then what is the use of saying we have teaching materials yet those materials are not suitable or are meant for a certain group of learners, I do not think is fair for LVIs”* (Teacher T from school A). Similarly, reports that most of the children with visual impairments are left behind due to the lack of adequate teaching resources were made by some of the participating learners; Learner 1 from school A: “ … .As it appears in our collage here, that we have placed books, one of the critical issues in my school is a tiny font used in learners’ textbooks,” referring to collage [Figure 1]. Learner 2 from school A, added: “The textbooks are written in a tiny font that causes us to not seeing what it has been written leading to our failure” These excerpts show that the participants found lack of appropriate support materials as constraining factors not the inclusion of LVIs in the catholic school classrooms.

### Inaccessible school classrooms

Participants’ verbatim statements showed that inaccessibility of school classrooms posed as a significant challenge to LVIs that they have necessary facilities that do not cater to the needs of LVIs. They highlighted that their country Lesotho as one of the developing countries, especially in impoverished communities; there is an absence of infrastructure that provides students with disabilities with the necessary facilities according to their individual’s disabilities. Furthermore, the participants also reveal that inaccessibility varies from class to class. The LVIs dominated the discussions and expressed their view by showing how their learning was affected by year-year. Learner 4 from school A narrated her school life experience: “ … *Some of the classrooms in my school have too much light, yes, I tried several sitting places in some classes, but because of too much light coming through the big windows, I did not see clearly*”. Learner 7 from school B: went ahead to exemplified a sad story of her school life: *“ … I even repeated a class due to my teacher’s negligence and negative impact of light from the big windows.”*

## Improving and managing rural school and community assets

The majority of the participants identified more constraining factors than enabling factors on the usage of their rural school assets that show the need to improve their fragmented usage to improve the well-being of LVIs. Evidence from this statement is the need for respect for LVIs in their learning environment. Some participants revealed that LVIs need to be valued, respected, and equally helped in order for them to gain access to education. Teacher C from school B noted: “ … *one of the positive aspects is the way we have to work with LVIs; I have found out that they are human beings like everyone and need to be respected***,”** Teacher S from school A also mentioned: *“You know! Working with LVIs demand change of attitude”* It was clear from the participants’ point of view that, interpersonal relationships as a factor that enables the use of available assets (as shown in collages Figures 1–4) in their rural school context needed to be improved. Teacher C from school A highlighted the need for collaboration among the education stakeholders as a factor on which the participants draw towards the improvement of the well-being of LVIs within their rural school context by saying: *“ … ehmm I think, working collaboratively with SGBs will enable school facilitation and management with teachers and enable the voices of parents to be heard on behalf of their children with SEN.”* Teacher C’s ideas supplemented the idea that LVIs needs to be respected and valued.

## Discussions

In this current study, the focus was on the improvement of the well-being of LVIs in rural schools. The improvement of LVIs’ well-being may be very challenging; however, everybody’s concern, yet manageable, requiring planning in realizing their potentials. Recognition that rural schools and rural communities possess resources, with both enabling and constraining factors in their usages to improving the well-being of LVIs and that there are more constraining factors towards the usage of the available assets and that if not considered are likely to hinder the teaching and learning for LVIs and as a result could become barriers for the improvement of their well-being. Evidence of the above claims is relevant to asset-based theory (Kretzmann & McKnight, 1993), where Kretzmann and McKnight, posit that community’s development and empowerment takes place from the inside out using the existing assets, resources, and abilities of the individual and the community. LVIs are also identified as active agents of change (Dunst, Raab, & Hamby,, 2017). Myende (2014) stated that the nature of solving individual and community problems required tapping into their inherent strengths. According to Dunst et al. (2017) to appropriately improving or enhancing the well-being of LVIs within their environments, requires the active involvement of LVIs regarding decisions concerning them. Similarly, Ebersöhn and Eloff (2006) established that the improvement of the well-being of LVIs requires the collaborations of the school-community support and sharing of assets.

In this study, one of the essential points of departure for the improvement of the well-being of LVIs was the understanding and identification of the available resources. The results of this study showed that these assets have a positive impact on learning and the improvement of the well-being of LVIs. The findings revealed that proper use of the existing assets could improve all learners’ academic performances, improve the well-being of LVIs, and promote collaborative learning. However, during the analysis of their collages, the participants, especially learners (LVIs), further indicated that their needs are overlooked and ignored, leading to other findings from the participants, which showed that the identified assets could have either enabling or constraining factors on their usage. This shows that the participants had a realistic view concerning the enabling factors on the usage of the existing assets within their rural school context.

These enabling factors created an opportunity for the enablement of LVIs’ involvement in the decision making and strengthened relationships among the education stakeholders within their rural schools. Besides, participants indicated that LVIs felt a sense of belonging as they interact with others and have a say in the issues concerning them. Their enhanced learning condition enable academic networking through which LVIs felt welcomed, grew, and developed holistically. On the other hand, the study’s findings also revealed that the participants identified constraining factors in the usage of the identified asset within their rural school. These constraints pose a significant challenge to the LVIs’ learning as it negatively affected their SEN and their well-being. One of those constraining factors which were also confirmed by works of literature is the shortage of special education teachers in rural schools (Myende & Hlalele, 2018). For these participants, the shortage of qualified specialist teachers could hinder the educational performances of individuals with “SEN.” Therefore, as a way of addressing this challenge, empowerment for all agents was identified during the analysis of the collages.

Therefore, the findings of this study showed the need for the improvement of resources within their schools that were made available to enhance teaching and learning. It is evident that in this study, that the use of available assets has constraining factors that need various measures to be taken by the rural schools and communities to enhance LVIs’ well-being based on the findings of this study. Therefore, the study recommends realization and understanding of the unique needs of LVIs, based on the fact that their needs draw on everybody’s concern. In this regard, this study sought to allow LVIs to reflect on how their well-being could be improved. The most critical point of departure is that the participants (teachers and learners) considered themselves as active agents, not passive objects; therefore, they need to be heard and recognized in the decisions concerning them.

## Strengths and limitations

Ensuring trustworthiness in a qualitative research study is essential as an indicator of the reality of the researcher’s data analysis, findings and conclusion (Maree, 2017). In a qualitative research project, trustworthiness is characterized by, credibility, transferability, dependability and the conformability of a study (Shenton, 2004). For this reason, trustworthiness becomes an important aspect in this study through the researchers’ accountability to produce the results that are worth paying attention to and useful to the readers in the field related to the current research project (Coleman, 2017). Therefore, to guarantee the honesty of this research project, the researchers adhered to the pillars of trustworthiness linked to the strengths and weakness of this research. According to Anney (2014), credibility is referred to as the trustfulness of the study by measuring what it is supposed to measure. To ensure that the study is credible, the researchers spent reasonable period of time on the field to become familiar with the research project.

Another important aspect to ensure the truthfulness of this study is transferability which is referred to the degree to which the research findings can be transferred to other settings and contexts (Coleman, 2017). In this study, the researchers ensured transferability by incorporating multiple data generation methods (Collage creation activities and focus group discussions) to increase confidence in the research findings. Additionally, the researchers employed proper procedures to ensure that the study achieved dependability by revisiting the research sites to verify from the participants that the information recorded during data generation process was the true reflection of what they have said (Shenton, 2004). The study comprised a sample of 28 participants and the special education teachers’ ratio was very small as compared to general education teachers within the selected rural schools in Lesotho; therefore, it was the researchers’ duty to be very careful in collage creation groups and focus group discussion strategies not to allow bias from minority number of specialist teachers, thus ensuring conformability as the process that establishes that the study is free of bias during the process of data generation and interpretation of results (Coleman, 2017). The findings of this research contribute positively to the knowledge base around improving the well-being of LVIs in rural schools. However, these findings cannot be generalized to all LVIs in other contexts and settings because the use of an asset-based approach may seem to vary cross-culturally due to differing expectations (Hlalele, 2012). As a result, future research should be conducted in further contexts to ensure the transferability of the current findings.

## Implications

Historically, LVIs’ voices have been ignored. However, the participants of the present study within rural schools in Lesotho viewed the right of all learners, including LVIs, to education as equal to other learners and that these needed to be valued as such. There was a severe need for management of resources available in Lesotho rural schools to create unique inclusion systems to cater for SEN and the well-being of LVIs. The participating schools could gain insight into and reflected on the available assets within the school to improve the well-being of LVIs. The teachers could furthermore gain insight into learners’ perceived assets and hopefully incorporate them into their decision-making processes, as these are vital and sometimes overlooked. Findings from this study will hopefully help future researches and policymakers to advocate for and design programmes that will target the specific needs of LVIs.

## Conclusion

Active involvement of LVIs is relatively new, emerging topic in Lesotho. This study sought to understand how the primary schools in the rural areas of Lesotho could improve the well-being of LVI using the asset-based approach. The study managed to elucidate the existing assets, and identified that in the usage of the existing assets, there were both constraining and enabling factors towards the improvement of the LVI’s holistic well-being. It was also important to work with LVIs in this study because the asset-based approach positioned them not just as victims or passive learners but also as agents who can make a meaningful contribution in their lives and in their schools. Their views call for a collaborative work to promote healthy interpersonal relationships and shared learning; academic networking whereby all agents (parents, teachers, SGBs, nurses, and learners including LVIs) work together, sharing ideas, and bringing solutions. This study may ignite dialogue among different education stakeholders on how best they can work together to remove the constraints in the usage of the existing assets, and at the same time also help to come up with innovative ideas to improve the well-being of LVIs in schools and beyond. Having done this study, we believed that participatory and visual methods such as collages create a space for other education stakeholders to construct, rethink, and reconstruct different discourses that will possibly improve the health and well-being of learners from all walks of life.
